# Infrared Light Emission Devices Based on Two-Dimensional Materials

**DOI:** 10.3390/nano12172996

**Published:** 2022-08-30

**Authors:** Wenyi Li, Hui Li, Karim Khan, Xiaosong Liu, Hui Wang, Yanping Lin, Lishang Zhang, Ayesha Khan Tareen, S. Wageh, Ahmed A. Al-Ghamdi, Daoxiang Teng, Han Zhang, Zhe Shi

**Affiliations:** 1 School of Physics & New Energy, Xuzhou University of Technology, Xuzhou 221018, China; 2 Shenzhen Engineering Laboratory of Phosphorene and Optoelectronics, Collaborative Innovation Center for Optoelectronic Science and Technology, Key Laboratory of Optoelectronic Devices and Systems of Ministry of Education and Guangdong Province, Shenzhen University, Shenzhen 518060, China; 3 School of Electrical Engineering & Intelligentization, Dongguan University of Technology, Dongguan 523808, China; 4 School of Mechanical Engineering, Dongguan University of Technology, Dongguan 523808, China; 5 Department of Physics, Faculty of Science, King Abdulaziz University, Jeddah 21589, Saudi Arabia

**Keywords:** 2D materials, infrared emission, spontaneous emission, laser

## Abstract

Two-dimensional (2D) materials have garnered considerable attention due to their advantageous properties, including tunable bandgap, prominent carrier mobility, tunable response and absorption spectral band, and so forth. The above-mentioned properties ensure that 2D materials hold great promise for various high-performance infrared (IR) applications, such as night vision, remote sensing, surveillance, target acquisition, optical communication, etc. Thus, it is of great significance to acquire better insight into IR applications based on 2D materials. In this review, we summarize the recent progress of 2D materials in IR light emission device applications. First, we introduce the background and motivation of the review, then the 2D materials suitable for IR light emission are presented, followed by a comprehensive review of 2D-material-based spontaneous emission and laser applications. Finally, further development directions and challenges are summarized. We believe that milestone investigations of 2D-material-based IR light emission applications will emerge soon, which are beneficial for 2D-material-based nano-device commercialization.

## 1. Introduction

Infrared (IR) emission is commonly defined as the electromagnetic wave range from 0.76 to 300 μm [1]. In sharp contrast to that of visible emission, IR emission plays a key role in both military and civilian applications, including night vision [2,3], remote sensing [4,5], surveillance [6,7], target acquisition [8,9], optical communication [10,11], and so forth [12,13,14,15]. Motivated by this, numerous efforts have been made in the past few decades [16,17]. However, the generation, detection, and manipulation of IR emission based on conventional semiconductors and solid-state materials suffer some severe challenges, such as low generation efficiency [18,19], request of cooling system [20,21], low response speed [22,23], and single generation or detection band [24,25]. In order to take full advantage and obtain a better insight into IR emission, novel materials need to be developed to meet the requirement of practical applications [26,27,28,29,30,31].

Since graphene was successfully synthesized in 2004 [32], plenty of two-dimensional (2D) materials have been developed, including black phosphorus (BP) [33,34], transition metal dichalcogenides (TMDCs) [35,36], topological insulators (TIs) [37], and so forth [28,38,39,40,41,42,43,44,45,46,47,48,49,50,51,52]. These materials have attracted considerable attention in photonics and optoelectronic fields due to their advantageous properties, such as high room temperature carrier mobility [53,54], tunable bandgap [55,56], fast response speed [57,58], ultra-broadband optical response, and absorption [59,60]. Based on these fascinating properties, some representative progresses about IR technology have been exhibited, which prove that 2D materials hold great potential for high-performance IR photonics and optoelectronic applications [61,62,63,64]. Additionally, combined with doping [39,65], heterostructures [66,67], and plasmon techniques [68,69], the properties of these materials can be efficiently modified, which further enhance the potential of 2D-material-based IR photonics and optoelectronic applications for IR light emission device applications in particular. Recently, some reviews reported 2D-material-based IR optoelectronic devices [1,26,30], which are mainly focused on the synthesis, IR modulator, photodetector, and light emission device applications. As an emerging next generation optoelectronic device, IR light emission devices, and spontaneous IR light emission and laser devices in particular, need to be further investigated. In this regard, comprehensive and detailed understandings of 2D-material-based IR light emission devices are crucial for their further development. Motived by this point, we summarized the recent progress in the field of 2D material IR light emission device applications, including the suitable 2D materials and recent progress of 2D materials in light emission. Finally, a perspective on future research and challenges of these fascinating materials is also proposed [70,71,72,73,74].

## 2. 2D Materials Candidates

According to density functional theory (DFT) calculations, nearly 5619 compounds possess layered structures and 1825 compounds can be potentially or easily exfoliated [75]. In terms of IR light emission applications, a strong light–matter interaction with IR electromagnetic wave is required. To satisfy this demand, the bandgap of 2D materials should be smaller than the incident light. Consequently, the amount of suitable or available materials is reduced significantly. Thus, it is of great significance to search or develop novel 2D materials with excellent optoelectronic properties in the IR range. In this section, we briefly introduce 2D materials candidates that are suitable for IR light emission applications, including their typical structures and properties.

### 2.1. Graphene

Graphene is the most investigated 2D material since it was discovered in 2004 [32]. Numerous investigations proved that graphene possesses a fine-structured constant determined absorption of απ ≈ 2.3%, ultra-high room temperature electron mobility (up to 2.5 × 10^5^ cm^2^ V^−1^ s^−1^), and strong light–matter interaction strength [76]. Additionally, graphene features, such as being 2D in nature and having semi-metallic properties [77,78], mean that the free charges are smaller than that of metals and the electrical properties of graphene can be modified via solid electrolyte gating or doping means. Thus, graphene can be considered a versatile material for diverse applications.

In terms of IR light emission applications, the light–matter interaction plays a determinant role. Fortunately, absorption of monolayer graphene possesses an ultra-high absorption efficiency, which was predicted to be 2.3 ± 0.2% per layer from the visible to IR bands, ensuring that graphene holds great potential for light emission applications [32,76]. Meanwhile, through integration with plasmon or combined with metamaterials, the absorption efficiency can be further enhanced from the ultraviolet to microwave bands [79,80]. Consequently, this paves a way to realize high-performance light emission devices.

### 2.2. BP and Related Materials

BP was rediscovered in 2014 [33]. Its superior properties, such as high room temperature carrier mobility (up to 1350 cm^2^ V^−1^ s^−1^), tunable bandgap (0.3~1.5 eV), high ON/OFF ratio (10^5^), strong light–matter interaction, and so forth [65,81], enable this fantastic material to be applied in various application fields, including energy storage [82,83], optoelectronic devices [84,85], catalysts [86,87], biomedicine [88,89], and others [90,91]. Meanwhile, owing to the sp^3^ hybridization, monolayer BP exhibits a typical puckered structure along the x direction (armchair direction), leading to an anisotropic band structure; the corresponding thermal, electrical, and optical properties are predicted to be highly anisotropic as well [92].

Regarding IR light emission device applications, BP possesses a direct bandgap range from monolayer to bulk conditions, which is beneficial for realizing efficient light absorption behavior form the visible to IR bands, and the light–matter interaction can be efficiently enhanced. Particularly, via introducing extra modulation methods, such as high pressure, doping, mechanical strain, and quantum confinement, the bandgap of BP can be extended from 0.3~1.5 eV to 0~2 eV, which covers the whole IR wavelength band [39]. For instance, in sharp contrast to traditional and other dimensional materials, BP can easily be large-scale and efficiently synthesized through exfoliation means [93], which greatly enhances its potential for high-performance photoelectric device applications. Recently, black arsenic phosphorus (b-AsP) and black phosphorus carbide (b-PC) have drawn considerable attention due to their high light–matter interaction with IR light [94,95]. By tuning the chemical composition of these two compounds, the bandgap can be precisely tuned, and the absorption IR wavelength can be extended to 14 μm, which is much larger than that of pristine BP. These advantageous properties ensure that BP and BP-related materials hold great potential for IR light emission device applications.

However, the environmental instability of BP under ambient conditions, which is caused by the degradation of BP in air through the cooperation between oxygen-, water- and light-induced oxidation, strictly hinders its further application [66]. To overcome this severe challenge, many attempts have been performed. Remarkably, doping and modification by metal ion are two main processes that can significantly improve the environmental stability.

### 2.3. Transition Metal Dichalcogenides

TMDCs are usually defined by a 2D layered structure in the form of X–M–X, and the chemical formula is MX_2_, where M is a transition metal element and X is a chalcogen [35]. Through the weak van der Waals interaction, the adjacent layers are held together. In sharp contrast to that of BP, the bandgap of TMDCs vary from direct to indirect behaviors as the thickness increases from monolayer to multilayer. The bandgap of TMDCs range from 1.0 to 2.0 eV, which means TMDCs are suitable for near-infrared (NIR) photoelectric applications [96,97]. Moreover, at resonance exciton wavelengths, the absorption of monolayer TMDCs is larger than 10%, which enables a strong light–matter interaction [98]. Remarkably, some investigations have proved that, via the introduction of some extra modulation processes, such as electrical field modulation [99] and defect engineering [100,101], the bandgap of TMDCs can be significantly suppressed, even in the long infrared (LIR) region. 

More recently, some noble TMDCs are successfully synthesized, such as PdSe_2_ [102,103] and PtSe_2_ [104,105]. These materials have drawn considerable attention due to their excellent properties, including narrow bandgap (0.3 eV for PdSe_2_ and 1.2 eV for PtSe_2_), extraordinary environmental stability, tunable conductivity, and so forth. Furthermore, TMDCs present ultrahigh surface atoms without dangling bonding [106,107], providing extra opportunities to modify their chemical and physical properties, which are particularly important for IR light emission applications.

### 2.4. Other 2D Materials

Apart from the materials mentioned above, some other 2D materials are suitable for IR light emission applications. Recently, PbSe [108,109] and CdSe [110,111] have aroused considerable attention due to their large absorption cross-sections, polarized optical properties, narrow bandgap, and controllable synthesis [112]. Combined hexagonal boron nitride (hBN) with surface plasmon polaritons is another effective approach to realize strong light–matter interaction, which can extend the absorption wavelength to mid-infrared (MIR) band [113]. Topological insulators (TIs), Bi_2_Se_3_ and Bi_2_Te_3_ in particular, are thought to be promising candidates for IR light emission applications due to their small bandgap, ultra-broadband and spectra absorption, higher carrier mobility and stronger light–matter interaction than that of graphene, which promote the development of TI-based IR light emission applications [114,115,116,117]. The In-based main group of metal chalcogenides, such as InSe, has been employed to fabricate IR photoelectronic devices owing to its tunable bandgap, weak electron-phonon scattering, high environmental stability, and extraordinary optical properties [118,119,120,121].

To facilitate a better understanding of the spectral range of some representative materials, a comparison of the operation spectra range of materials is listed in Table 1.

## 3. Spontaneous Emission

2D materials have been considered promising candidates for high-performance spontaneous emission applications due to their quantum confinement effect [122,123,124,125], which enable 2D materials that possess advantageous optical properties, including layer-dependent bandgap, spin–valley correlation [126,127], and large exciton-binding energy [128,129]. In this section, we briefly introduce some representative progress in 2D-material-based IR spontaneous emission applications, such as graphene, BP, TMDCs, and other 2D-material-based IR single photon emitters, photoluminescence (PL) generation, light emission devices (LEDs), tunable light emission, and so forth.

### 3.1. Graphene-Based Spontaneous IR Emission

**Figure 1:**
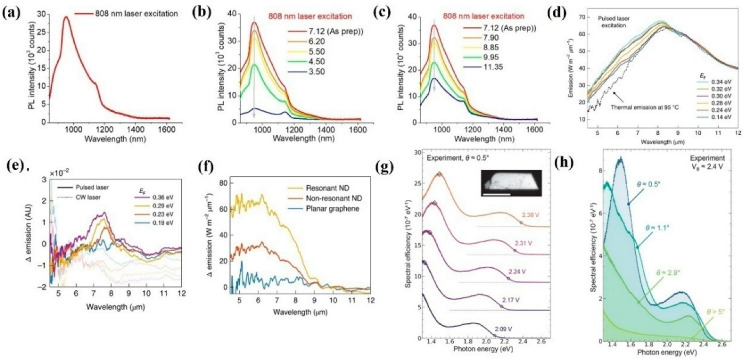
Light emission and devices based on graphene. (**a**) NIR emission of RGQD under 808 nm lased illumination. (**b**,**c**) Fluorescence spectra of RGQDs under different PH values and 808 nm lased illumination. (**d**) The Fermi–Level–dependent emission spectra of graphene. (**e**) The Fermi–Level–dependent emission spectra of graphene under pulsed/CW illumination. (**f**) The emission spectra of graphene with and without ND decoration. (**g**,**h**) The emission spectra of graphene/h-BN/graphene tunnel junctions under various bias voltages and twist angles, respectively. (**a**–**c**) Reprinted from [130], with permission from IOP Publishing Ltd., 2021; (**d**–**f**) Reprinted from [131], with permission from Springer Nature, 2021; (**g**,**h**) Reprinted from [132], with permission from American Chemical Society, 2021.

As aforementioned, graphene holds various advantageous properties, which prove that graphene can meet various demands of different applications. Due to this, graphene is also employed to realize high-performance spontaneous IR emission applications. Very recently, Naumov et al. demonstrated NIR light emission based on graphene quantum dots (QDs) [130]. As shown in Figure 1a, despite the generated light emission range from 900 nm to 1100 nm, less than 808 nm laser excitation can be attributed to the localized defect states caused by the hypochlorite reaction. Additionally, to acquire a better insight into the emission mechanism, the generated NIR emissions of the prepared graphene QDs under different pH value solutions are investigated. As presented in Figure 1b,c, as the pH varied from 3.50 to 11.32, the NIR emission intensity monotonously increased and decreased, respectively, which is caused by the excited state protonation and deprotonation of multiple species or reversible loose aggregation leading to several nonradiative pathways. All of these outstanding findings indicate that graphene QDs can be employed to realize high performance NIR light emission applications for biological and imaging applications in particular. Subsequently, combined with plasmons technique, Atwater et al. proved that the generated emission spectra wavelength can be effectively extended to the MIR band [131]. In the spectra range from 4 to 8 μm, the intensity of the emission monotonously increased as the Fermi Level of the graphene varied from 0.14 to 0.34 eV. In contrast, in the spectra range from 8 to 11 μm, the emission intensity monotonously decreased as the Fermi-level of the graphene varied from 0.14 to 0.34 eV (Figure 1d), which is mainly caused by the photoexcited charge carrier life time-reduced effect. To reveal the effect of pulsed and continuous wave (CW) excitation laser sources, the generated MIR emission as a function of pulsed and CW laser sources to different graphene Fermi Levels was performed. As shown in Figure 1e, the MIR emission under pulsed laser excitation is much higher than that of the CW condition, which can be ascribed to the origin of plasmons that contribute to MIR emission under pulsed laser excitation, is distinctly different from that of thermal plasmons. To further enhance the emission efficiency, nanostructures such as gold nanodisks (NDs) with and without resonant laser excitation wavelengths, are deposited onto the surface of the employed graphene; it can be clearly seen in Figure 1f that the MIR emission can be significantly enhanced with resonant NDs in the whole emission spectra range, which paves a way to realize higher efficiency for MIR emission applications. Apart from the above-mentioned means, forming graphene/h-BN/graphene tunnel junctions is another effective way to enhance the emission efficiency. As a result of this, Novotny et al. fabricated twist-controlled resonant light emission devices based on graphene/h-BN/graphene tunnel junctions [132]. Figure 1g reveals the emission efficiency as a function of various bias voltages with a 0.5° twist angle. The emission efficiency is proportional to the applied bias voltage. Under a constant applied bias voltage (2.4 V), the emission efficiency as a function of different twist angles are illustrated in Figure 1h; the spectral peak broadened as the twist angle increased from 0°to 5° and disappeared at 2.9°, which is caused by the twist-angle-dependent momentum mismatch between the Dirac cones. Remarkably, the tunable emission peak with applied bias voltage is beneficial for realizing high-performance integrated photonics and optoelectronic device applications.

### 3.2. BP-Based Spontaneous IR Emission

**Figure 2:**
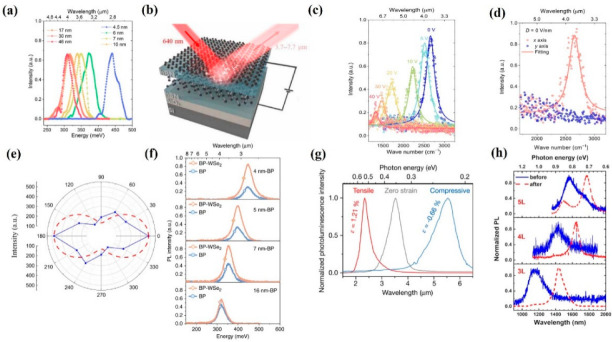
Light emission and devices based on BP. (**a**) The PL spectra as a function of various BP thicknesses. (**b**,**c**) The schematic and measured PL spectra of the device, respectively. (**d**) The PL spectra of BP under displacement fields of 0 V/nm. (**e**) The polarization−resolved EL spectra of the device. (**f**) The PL spectra of the device with various BP nanoflake thicknesses. (**g**) The PL intensity of BP with various applied strain. (**h**) The PL intensity of BP with and without annealing treatment. (**a**) Reprinted from [133], with permission from American Chemical Society, 2019; (**b**−**e**) Reprinted from [134], with permission from Wiley−VCH, 2020; (**f**) Reprinted from [135], with permission from American Chemical Society, 2020; (**g**) Reprinted from [136], with permission from Springer Nature, 2021; (**h**) Reprinted from [137], with permission from American Chemical Society, 2021.

BP possesses a thickness-dependent direct bandgap range from 0.3 to 2.0 eV, which covers the visible and MIR bands. Thus, BP holds great potential for light emission applications. As a result, numerous efforts have been drawn to realize high-performance light emission devices. Xia et al. demonstrated bright photoluminescence (PL) emission based on various BP nanoflake thicknesses ranging from 4.5 to 46 nm [133]. As the thickness of BP decreased, the frequency of PL spectra exhibited an obviously blue shift behavior, which is due to the increasing bandgap, suggesting that BP can be employed as a promising candidate for MIR light emission applications, as shown in Figure 2a. Subsequently, Yang et al. demonstrated that tunable MIR light emission based on consistently thick BP nanoflakes can be realized via applying various bias voltages and external displacement fields [134]. As shown in Figure 2b,c, by utilizing 640 nm laser excitation, the generated PL ranged from 3.7 μm to 7.7 μm when the applied bias voltages varied form 0 V to 40 V. Moreover, the polarization-resolved PL of BP under different displacement fields was investigated to reveal the anisotropic effect of BP. As shown in Figure 2d, the generated PL spectra exhibited a distinct linear polarization behavior under 0 V/nm displacement fields. Further increasing the displacement fields to 0.12 and 0.24 V/nm, the PL spectra presents a distinct linear polarization behavior as well, which is mainly due to the anisotropic optical conductivity near the band edge of BP and the optical inter-band transitions are permitted along the x direction. To further investigate the polarized light emission of BP, Kéna-Cohen et al. introduced an MIR emission device based on BP [135]. As illustrated in Figure 2e, the intensity of the generated electroluminescence (EL) spectra possesses a polarization ratio of ∼3 for the armchair (due to sp^3^ hybridization, a single layer of BP shows a puckered structure along the along the x axis, orthogonal to the corrugation direction) and zigzag (due to sp^3^ hybridization, a single layer of BP shows a puckered structure along the along the *y* axis, parallel to the corrugation direction) directions, indicating that the luminescence is indeed more polarized along the armchair direction than that of zigzag direction. Van der Waals heterostructures, which best combine various 2D materials at an artificial atomic level, provide extra opportunities to realize high-performance optoelectronic devices, such as MIR light emission devices. Recently, Chen et al. presented novel tunable MIR light emission devices based on BP and WSe_2_ heterostructures. As shown in Figure 2f, compared to that of pristine BP, the generated MIR PL intensity is much higher in BP and WSe_2_ heterostructures. Remarkably, a maximum enhancement of ~200% was achieved, suggesting great potential for BP-based heterostructures for high efficiency MIR light emission applications. Compared to the above-mentioned employed technique to modify the bandgap of BP, applied external strain is a simple and effective means,\ that can avoid complex fabrication processes. Based on this, Javey et al. demonstrated a tunable room temperature MIR light emission device based on BP with external strain treatment [136]. As illustrated in Figure 2g, with 0.66% and 1.21% strain along the zigzag direction, the PL spectra exhibit blue shift and red shift compared to that of pristine BP nanoflakes, indicating the strain-sensitive nature of BP. To enhance the stability of PL, Ning et al. performed an efficient IR light emission device based on thermal annealing BP nanoflakes [137]. As shown in Figure 2h, after thermal annealing treatment, the generated PL presents a red shift and line width narrowing behavior for all three different thicknesses of the BP nanoflakes, which is caused by the etching and annealing processes and non-uniform thickness of the original exfoliated thick BP. All of these outstanding findings suggest that BP is suitable for tunable, stable, and effective light emission applications.

### 3.3. TMDC-Based Spontaneous IR Emission

**Figure 3:**
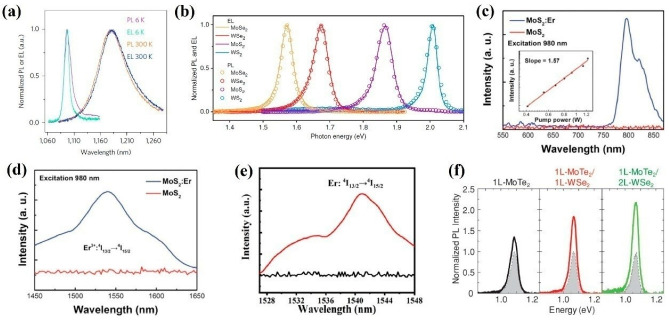
TMDC-based light emission and devices. (**a**) The PL and EL spectra of bilayer MoTe_2_. (**b**) The PL and EL spectra of monolayer MoSe_2_, WSe_2_, MoS_2_, and WS_2_. (**c**,**d**) The UC and DC PL spectra of MoS_2_, respectively. (**e**) The DC PL spectra of WSe_2_ with and without Er−doped treatment. (**f**) The PL spectra of 1L−MoTe_2_, 1L−MoTe_2_/1LWSe_2_, and L−MoTe_2_/2L−WSe_2_. (**a**) Reprinted from [138], with permission from Springer Nature, 2017; (**b**) Reprinted from [139], with permission from Springer Nature, 2018; (**c**,**d**) Reprinted from [140], with permission from Wiley-VCH, 2016; (**e**) Reprinted from [141], with permission from Elsevier, 2021; (**f**) Reprinted from [142], with permission from Wiley-VCH, 2018.

Multilayer TMDCs that possess indirect band structures have a negative contribution to the photoelectric conversion efficiency. Thus, most investigations on spontaneous emission of TMDCs are focused on mono- and bilayer TMDCs. Recently, Herrero et al. proved that bilayer MoTe_2_ can be utilized to realize high-performance integrated IR light emission devices [138]. To enable EL generation, a novel local gate was designed to form an ohmic contact p–n junction. As shown in Figure 3a, the PL and EL peaks were located at 1175 nm at room temperature; as the temperature decreased to 6 K, both the PL and EL peaks significantly narrowed and exhibited a blue shift behavior. To further improve the emission stability, Schottky contact is commonly employed. Recently, Javey et al. fabricated a simple and effective Schottky contact IR light emission device based on monolayer MoSe_2_, WSe_2_, MoS_2_, and WS_2_ [139]. Applying an AC voltage between the semiconductor and the gate, the EL could be achieved. As shown in Figure 3b, the measured EL emission was well consistent with the PL emission. This simple structure of the device paved an effective way to realize practical applications. Meanwhile, extending the emission wavelength to the telecommunication band is of great significance, which can significantly extend the application fields of TMDC-based light emission devices. The doping process is a conventional method that can meet this demand. Motivated by this, Hao and Xu et al. utilized Er-doped MoS_2_ and WSe_2_ to extend the emission band to 1550 nm, respectively [140,141]. Compared to pristine MoS_2_ and WSe_2_ nanoflakes, IR emission around 1550 nm from doped MoS_2_and WSe_2_ can be observed (Figure 3d,e), which correspond to ^4^I_13/2_→^4^I_15/2_ transitions of Er ions. Moreover, the upconversion (UC) PL spectra of MoS_2_ with and without Er-doped treatment exhibited an evident difference, as shown in Figure 3c. A PL peak at 800 nm of Er-doped MoS_2_ can be found, indicating that the doping process is an effective means to extend the emission wavelength. Apart from doping process, as aforementioned, the heterojunction technique is another commonly used method to enhance device performance. Based on this, Matsuda et al. fabricated atomically thin monolayer MoTe_2_- and WSe_2_-based heterostructures to enhance the intensity of IR light emission [142]. As illustrate in Figure 3f, compared to monolayer MoTe_2_, the PL intensity can be largely enhanced by using MoTe_2_ and WSe_2_ heterostructures, which is due to the flow of photogenerated electron–hole pairs from the WSe_2_ layer to the MoTe_2_ layer, contributing to the enhancement of PL intensity in the Type I MoTe_2_ and WSe_2_ heterostructure. All of these outcomes suggest that TMDCs hold great potential for IR light emission applications.

### 3.4. Other 2D-Material-Based Spontaneous IR Emission

**Figure 4:**
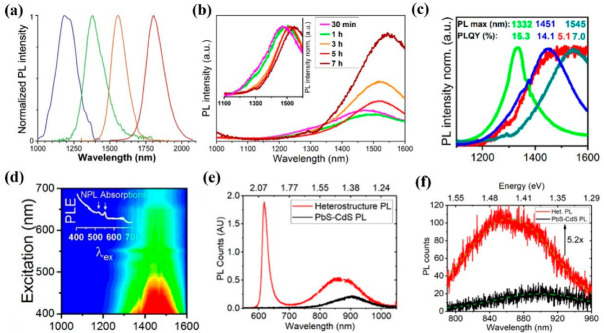
Light emission based on other 2D materials. (**a**) The normalized PL spectra of PbSe with different sizes. (**b**,**c**) The PL spectra of PbSe nanoplatelets with different reaction times and different PbSe populations, respectively. Inset of (b): normalized PL spectra. (**d**) The PL map of PbSe/CdSe heterostructure. (**e**) The PL spectra of WS_2_−PbS−CdS heterostructure and PbS−CdS. (**f**) The PL spectra of bare substrate and PbS−CdS QD. (**a**) Reprinted from [143], with permission from American Chemical Society, 2003; (**b**,**c**) Reprinted from [144], with permission from American Chemical Society, 2019; (**d**) Reprinted from [112], with permission from American Chemical Society, 2016; (**e**,**f**) Reprinted from [145], with permission from American Chemical Society, 2021.

Apart from the above-mentioned 2D materials, 2D colloidal semiconductors have aroused considerable attention in IR light emission applications due to their advantageous properties, such as tunable bandgap based on size, efficient PL generation, simple synthesis process, and so forth. Among these 2D materials, 2D PbSe is promising for high-performance IR emission applications due to its suitable bandgap, high PL quantum yields (QYs), and efficient and low cost synthesis process, which promote it in practical and commercial IR light emission applications. In this regard, Klimov et al. demonstrated tunable IR light emission based on 2D PbSe nanocrystals (NCs) of various sizes [143]. The wavelength of PL emission can be efficiently modified by utilizing 2D PbSe NCs (1100 nm to 1700 nm) of various sizes, as shown in Figure 4a. Remarkably, the generated PL emission covers the telecommunication band, which further extends its application fields. Latterly, Eychmüller et al. proved that the wavelength of PL emission can be tuned by using PbSe nanoflakes of different thicknesses [144]. The generated PL ranges from 1100 nm to 1600 nm and a maximum PL intensity is concentrated at 1545 nm. Moreover, it can be seen in Figure 4b,c that three peaks are located at 1332, 1451, and 1545 nm, and the corresponding PL QYs are 15.3%, 14.1%, and 7.0%, respectively. These outcomes further confirm that PbSe possess a size- or thickness-dependent bandgap, which is beneficial for tunable IR PL emission applications. For bioimaging, LEDs, and single photon emitter applications a higher intensity PL is commonly required. To meet this demand and extend 2D-material-based light emission application fields, a Type I semiconductor heterostructure is considered to be an effective means. Very recently, Schaller et al. demonstrated that the IR PL emission can be significantly enhanced by utilizing zero-dimensional (0D) PbSe QDs and 2D CdSe nanoflake heterostructures [112]. The PL excitation spectra measurement was performed to characterize the emission performance, as demonstrated in Figure 4d, the PL is concentrated around 1550 nm, with a line width of about 200 nm, which is comparable to other available semiconductor QDs. Subsequently, Raoet al. investigated IR PL emission based on monolayer WS_2_ and PbSe/CdSe QDs heterostructures [145]. In sharp contrast to that of a PbSe/CdSe dot-on-plate sample, the intensity PL emission of PbSe/CdSe heterostructures is much higher. Meanwhile, the PL emission spectra range is much wider than PbSe/CdSe heterostructures (Figure 4e), suggesting that Type I semiconductor heterostructures are beneficial for high efficiency IR PL emission. To obtain a better insight into the PL emission mechanism, Figure 4f presents the PL emission from fabricated heterostructures and pristine PbSe/CdSe [146]. Compared to that of a pristine PbSe/CdSe condition, a 47 nm blue shift can be observed. Additionally, the PL intensity is about 5.2 times that of the pristine PbSe/CdSe, which is mainly caused by the energy funneling from the directly excited monolayer WS_2_. These outcomes indicate that 2D colloidal semiconductors and their heterostructures can significantly modify the IR PL emission and provide extra opportunities to meet the various demands of different applications.

To facilitate a better understanding of the 2D materials for LEDs operation, potential 2D material candidates are listed in Table 2.

## 4. Laser

Laser is considered one of the greatest inventions of the 20th century; it has drawn considerable attention due to its wide spread applications. During the rapid development of the semiconductor industry, nanoscale laser has attracted considerable attention. 2D materials, such as TMDCs, BP, and graphene, are employed as gain mediums due to their superior properties, including large exciton-binding energy, ultralow lasing threshold, tunable bandgap, and so forth. In this section, we briefly introduce some recent representative progresses in 2D-material-based IR laser applications.

**Figure 5:**
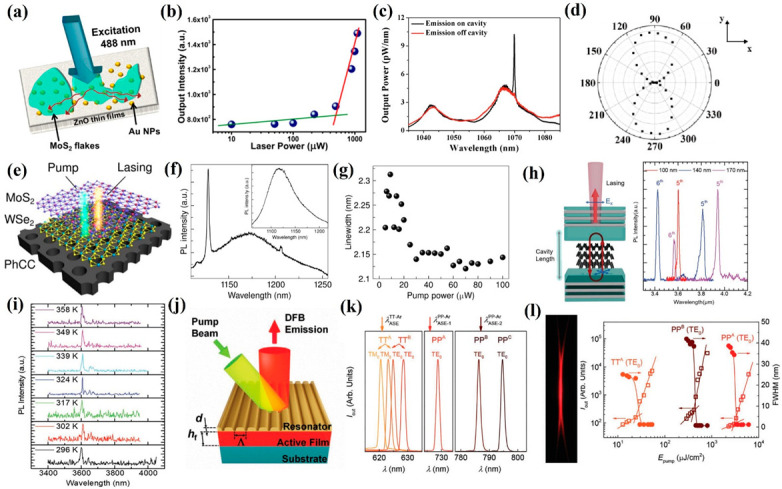
Laser based on 2D materials. (**a**) Schematic diagram of the MoS_2_-based laser device. (**b**) The lasing threshold of the multilayer MoS_2_-based device. (**c**) The measured laser emission of the MoTe_2_-based device with and without cavity. (**d**) The polarization-dependent measurement of the laser emission. (**e**) Schematic diagram of the MoS_2_-/WSe_2_-heterostructure-based device. (**f**) The measured laser emission of the WSe_2_ and MoS_2_ heterostructures with nanocavity. Inset: the laser emission without nanocavity. (**g**) The line width of the laser as a function of pump power. (**h**) Schematic diagram of the BP-based laser device and the measured laser emission of various BP thicknesses. (**i**) The laser emission temperature-dependent measurement of the device with a 140 nm thick BP. (**j**) Schematic diagram of the nanographene-based DFB laser device. (**k**) The measured laser emission of the device. (**l**) The optical image of the laser beam of the device and the intensity, with line width of the laser emission as a function of pump energy density. Reprinted with permission from (**a**,**b**) Reprinted from [146], with permission from American Chemical Society, 2018; (**c**,**d**) Reprinted from [147], with permission from The Korean Physical Society, 2018; (**e**–**g**) Reprinted from [148], with permission from American Association for the Advancement of Science, 2019; (**h**,**i**) Reprinted from [149], with permission from Wiley-VCH, 2020; (**j**–**l**) Reprinted from [150], with permission from Wiley-VCH, 2021.

In 2018, with combined multilayer MoS_2_ and Au NPs, Dixit et al. demonstrated an IR random laser range from 800 nm to 950 nm (Figure 5a) [146]. Meanwhile, the lasing threshold was measured to be 500 μW (Figure 5b), which is much lower than some commercial lasers. To further enhance the lasing intensity and suppress the line width, a cavity resonator is commonly applied. Recently, Li et al. fabricated a nanoscale laser based on MoTe_2_ with a silicon nanocavity [147]. Combined with a silicon nanocavity, a clear peak was located at 1070 nm and the corresponding Q-factor is 3627 (Figure 5c), indicating that the applied nanocavity is beneficial for lasing behavior. The polarization-dependent measurement was performed to further evaluate the lasing performance of the device, as illustrated in Figure 5d; the laser emission can only be measured along their direction, which represents the TE-like mode in the applied silicon nanocavity. For further expansion of the lasing wavelength, Gao et al. utilized MoS_2_/WSe_2_ heterostructures as gain materials in the laser system (Figure 5e) [148]. At room temperature, a sharp peak at 1128 nm with a line width of 2.15 nm can be observed (Figure 5f,g), which is slightly smaller than that of the 5 K condition and exhibits an enhancement of the Q-factor of the cavity. Apart from TMDCs, BP also can be used to fabricate IR lasers due to its aforementioned advantageous properties. Combined with an open distributed Bragg reflector (DBR) cavity (Figure 5h (left)), Pan et al. exhibited a wavelength tunable MIR laser based on BP nanoflakes [149]. Three-layer thick BP nanoflakes (100 nm, 140 nm, and 170 nm) were employed as the gain medium, and the wavelength of the laser emissions can be tuned from 3425 nm to 4068 nm (Figure 5h (right)), which correspond to the fifth- and sixth-order cavity modes. The thermal stability is another key parameter of the laser device, which has an important impact on its reliable operation. As described in Figure 5i, as the temperature varied from 296 K to 358 K, the lasing peak at 3600 nm presents a negligible frequency shift behavior, suggesting the great thermal stability of the device and enabling BP as a promising candidate for high-performance tunable MIR laser applications. Nanographene is another promising material that can realize IR nanoscale laser applications due to its size-dependent bandgap. For example, Díaz-García et al. successfully demonstrated nanographene-based NIR-distributed feedback (DFB) lasers (Figure 5j) [150]. For a PP-Ar chemical structure nanographene-based device combined with an employed DFBs structure, the generated laser emissions can extend the laser emission into the NIR band and can be concentrated at 729 nm and 795 nm (Figure 5k). For a TT-Ar chemical structure nanographene-based device, the profile of the laser beam with a divergence of ≈1.8 × 10^−3^ rad is shown in Figure 5l (left), suggesting that the propagation direction of the generated laser is perpendicular to its grating lines. To evaluate the laser threshold of the fabricated device, the line width of laser emissions as a function of incident laser energy density was performed, as shown in Figure 5l (right), and the threshold of the device ranges from 25 μJ cm^−2^ to 47 μJ cm^−2^, which is slightly smaller than that of other commercial DFB lasers. All of these outcomes indicate that 2D materials, such as TMDCs, BP, and graphene, are suitable and hold great potential for IR laser applications.

To facilitate a better understanding of laser operation based on 2D materials, a comparison of the pulse duration of several lasers and their thresholds is listed in Table 3.

## 5. Conclusions and Outlook

Because of their extraordinary optoelectronic properties, 2D materials are intensively employed to fabricate IR light emission devices. In this review, we investigated the recent progress of 2D-material-based IR light emission devices, including 2D material candidates that are suitable for MIR applications, such as graphene, BP, TMDCs, topological insulators, and other 2D materials, as well as their device application in the IR band: in particular, spontaneous IR emission and laser applications. Although several kinds of 2D materials are promising for IR light emission applications, opportunities and challenges still remain for researchers.

1. In terms of LEDs, compared to commercially available LEDs, the external quantum efficiency and operation stability of 2D-material-based LEDs are still too low. To satisfy the demand of practical applications, the external quantum efficiency and operation stability need to be significantly improved. Furthermore, the emitting wavelength needs to be further extended, as it is far from practical applications. For the driven mode, more attention should be concentrated on an electrically driven mode. Thus, it is of great significance to develop novel 2D-material-based LEDs with various configurations in the future.
2. Single-photon emitters based on 2D materials are thought to be originated from the defects. However, the underlying physical mechanism, excitation processes, and atomic structure are still under debate. Meanwhile, the emitting wavelength needs to be extended into the deep IR region.
3. The lasing threshold is relatively low at a lower temperature; however, for room temperature lasing, the threshold needs to be significantly suppressed. To satisfy the practical applications, an IR laser source with long operation stability, high peak intensity, and narrow line width at room temperature needs to be further developed.
4. To meet the demand of practical applications, novel 2D materials that possess a suitable bandgap, excellent environmental stability, long-term operation stability, and controllable synthesis need to be developed.

In conclusion, 2D-material-based IR light emission devices have already achieved some milestone achievements. However, these devices also face some severe challenges as aforementioned. Ongoing investigations into these advantageous materials in photonic and photoelectronic systems, including IR spontaneous emission and laser devices, is anticipated. Furthermore, a more comprehensive understanding of IR light emission devices based on 2D materials, including their properties, synthesis process, and photoelectric applications, needs continual investigation.

## Figures and Tables

**Table 1:** The spectral range of the operation of some representative materials.

2D Materials	Bandgap (eV)	the Spectral Range of Operation (μm)	Ref.
BP	0.3~1.5	0.83~4.13	[65]
b-AsP	0.15	8.27	[94]
b-PC	0.59	2.10	[95]
PbSe	0.413~1.77	0.70~3.00	[109]
CdSe	0.22~0.38	3.33~5.71	[111]
PdSe_2_	1.3	0.95	[102]
PtSe_2_	0.3	4.13	[104]
2D Te	0.35~1.265	0.98~3.54	[29]
Bi_2_Se_3_	0.21	5.90	[114]
Sb_2_Te_3_	0.45	2.75	[115]

**Table 2:** 2D material candidates for LEDs operation.

2D Materials	Bandgap (eV)	Wavelength (μm)	Luminous Mode	Ref.
Graphene QDs	1.13~1.38	0.90~1.10	photoluminescence	[130]
BP	0.30~1.50	0.83~4.13	photoluminescence	[133]
BP	0.16~0.34	3.70~7.70	photoluminescence	[134]
WSe_2_	1.0~2.4	0.51~1.24	electroluminescence	[139]
Er-doped MoS_2_	0.8	1.55	electroluminescence	[141]
MoTe_2_/Wse_2_	1.1	1.127	electroluminescence	[141]
PbSe	0.73~1.13	1.10~1.70	photoluminescence	[143]

**Table 3:** Laser operation based on 2D materials.

2D Materials	Wavelength (μm)	Pulse Duration	Threshold	Frequency	Ref.
Bi_2_Te_3_ deposited on CaF_2_	2.979	1.37 μs	3.39 μJ	81.96 KHz	[115]
Bi_2_Te_3_	2.8	1.3 μs	ND	92 KHz	[116]
Bi_2_Se_3_	3.0	1.5 μs	48 mW	55.1 KHz	[117]
MoS_2_	0.80~0.95	ND	500 μW	ND	[146]
MoTe_2_	1.07	ND	30 kW/cm^2^	ND	[147]
MoS_2_/WSe_2_	1.128	1.9 ps	54 μW	ND	[148]
BP	3.42~5.06	Quasi-CW	1.3 mJ/cm^2^	ND	[149]

## Data Availability

Not applicable.
